# Development of the novel GlyT1 inhibitor, iclepertin (BI 425809), for the treatment of cognitive impairment associated with schizophrenia

**DOI:** 10.1007/s00406-023-01576-z

**Published:** 2023-03-27

**Authors:** Holger Rosenbrock, Michael Desch, Glen Wunderlich

**Affiliations:** 1 Boehringer Ingelheim Pharma GmbH & Co. KG, Biberach an der Riss, Germany; 2 Boehringer Ingelheim Pharmaceuticals, Inc., 900 Ridgebury Road, Ridgefield, CT 06877 USA

**Keywords:** Iclepertin, BI 425809, GlyT1 inhibitor, Cognitive impairment, Schizophrenia, NMDA receptor

## Abstract

Schizophrenia is a psychiatric disorder characterised by symptoms in three domains: positive (e.g. delusions, hallucinations), negative (e.g. social withdrawal, lack of motivation) and cognitive (e.g. working memory and executive function impairment). Cognitive impairment associated with schizophrenia (CIAS) is a major burden for patients and negatively impacts many aspects of a patient’s life. Antipsychotics are the standard-of-care treatment for schizophrenia but only address positive symptoms. So far there are no approved pharmacotherapies for the treatment of CIAS. Iclepertin (BI 425809) is a novel, potent and selective glycine transporter 1 (GlyT1) inhibitor, under development by Boehringer Ingelheim for the treatment of CIAS. Phase I studies have shown it to be safe and well tolerated in healthy volunteers, and central target engagement (inhibition of GlyT1) was achieved in a dose-dependent manner from 5 to 50 mg in healthy volunteers. A Phase II study has demonstrated that iclepertin is safe and well tolerated in patients with schizophrenia and improves cognition at doses of 10 mg and 25 mg. Phase III studies are ongoing to confirm these initial positive safety and efficacy findings with the 10 mg dose, and if successful, iclepertin could become the first approved pharmacotherapy used to treat CIAS.

## Introduction

Schizophrenia is a complex, heterogeneous psychiatric disorder associated with a wide range of debilitating symptoms that affect daily functioning and may also contribute to reduced life expectancy [1–3]. In addition, individuals with schizophrenia may experience discrimination and social stigma as a result of their illness [4]. It is estimated that approximately 0.32% of the worldwide population, or approximately 24 million people globally, are affected by schizophrenia [4].

**Fig. 1:**
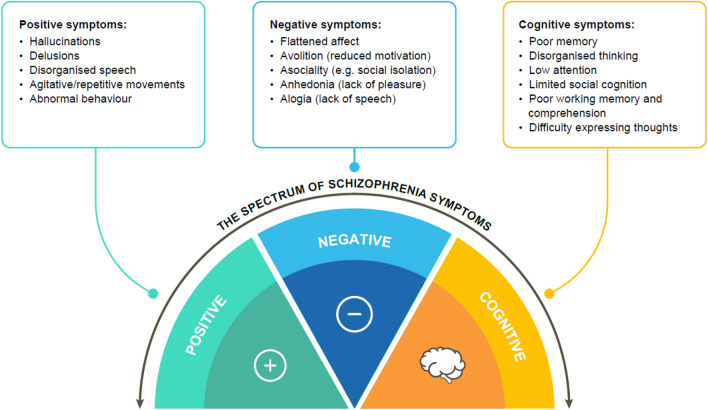
The three symptom domains of schizophrenia [2, 3]

The onset of symptoms most frequently occurs in late adolescence or the twenties [4]. Symptoms of schizophrenia span three domains: positive, negative and cognitive (Fig. 1), and symptoms may vary in severity between individuals [1–3]. Positive symptoms may include delusions and hallucinations as well as disorganised behaviour and speech, whereas negative symptoms are associated with social withdrawal, lack of motivation, decreased energy, loss of interest in normally enjoyable activities, a flattened affect and anhedonia (the inability to feel pleasure) [1–3]. Cognitive symptoms, which are often present before the onset of psychosis and remain after successful treatment of psychosis with antipsychotics, include impairments in working memory and executive function, difficulty expressing thoughts, and reduced processing speed [1, 3]. Cognitive impairment associated with schizophrenia (CIAS) is common and contributes to functional disabilities in everyday life [1, 3, 5]. Thus, CIAS can strongly impair the patient’s quality of life [1, 3, 5] and may lead to poorer functional outcomes overall [6]. Indeed, the degree of CIAS is now thought to be the best predictor of long-term functional outcomes for patients with schizophrenia [7].

Antipsychotic drugs, the gold standard (and only approved pharmacological option) for the treatment of schizophrenia, primarily address the positive symptoms, such as delusions and auditory hallucinations, but do not effectively treat negative symptoms or CIAS, or fully address daily functioning [8]. Currently, there are no approved pharmacotherapies specifically targeting CIAS [5]. Although clozapine is effective at improving positive and negative symptoms in patients who did not previously respond to conventional treatments, and is widely recommended for treatment-resistant patients [9–11], underutilisation of clozapine has been reported, resulting from treatment complexity, adverse effects and associated costs [12–15]. Consequently, there is an urgent clinical need to develop effective pharmacotherapies to improve CIAS and thereby potentially improve the quality of life and daily functioning among patients with schizophrenia.

## The role of glutamatergic signalling in CIAS

In the central nervous system, glutamatergic synapses are responsible for excitatory neuronal signalling in the cortex [16, 17]. When an action potential arrives at the presynaptic membrane, glutamate, the principal excitatory neurotransmitter in the brain, carries the signal across to the postsynaptic neuron [17, 18]. Glycine, another neurotransmitter released by both neurons and astrocytes, supports glutamatergic signalling [19]. Glycine has two major functions in the central nervous system: it acts as an inhibitory neurotransmitter in glycinergic neurons, primarily in the hindbrain [20] and also acts as a co-agonist for *N-*methyl*-*D*-*aspartate (NMDA) receptors in excitatory glutamatergic neurotransmission, primarily in the forebrain [19, 21]. The release and reuptake of glycine is controlled by the glycine transporter 1 (GlyT1), which is expressed in glial cells and presynaptically in neurons [19, 22].

**Fig. 2:**
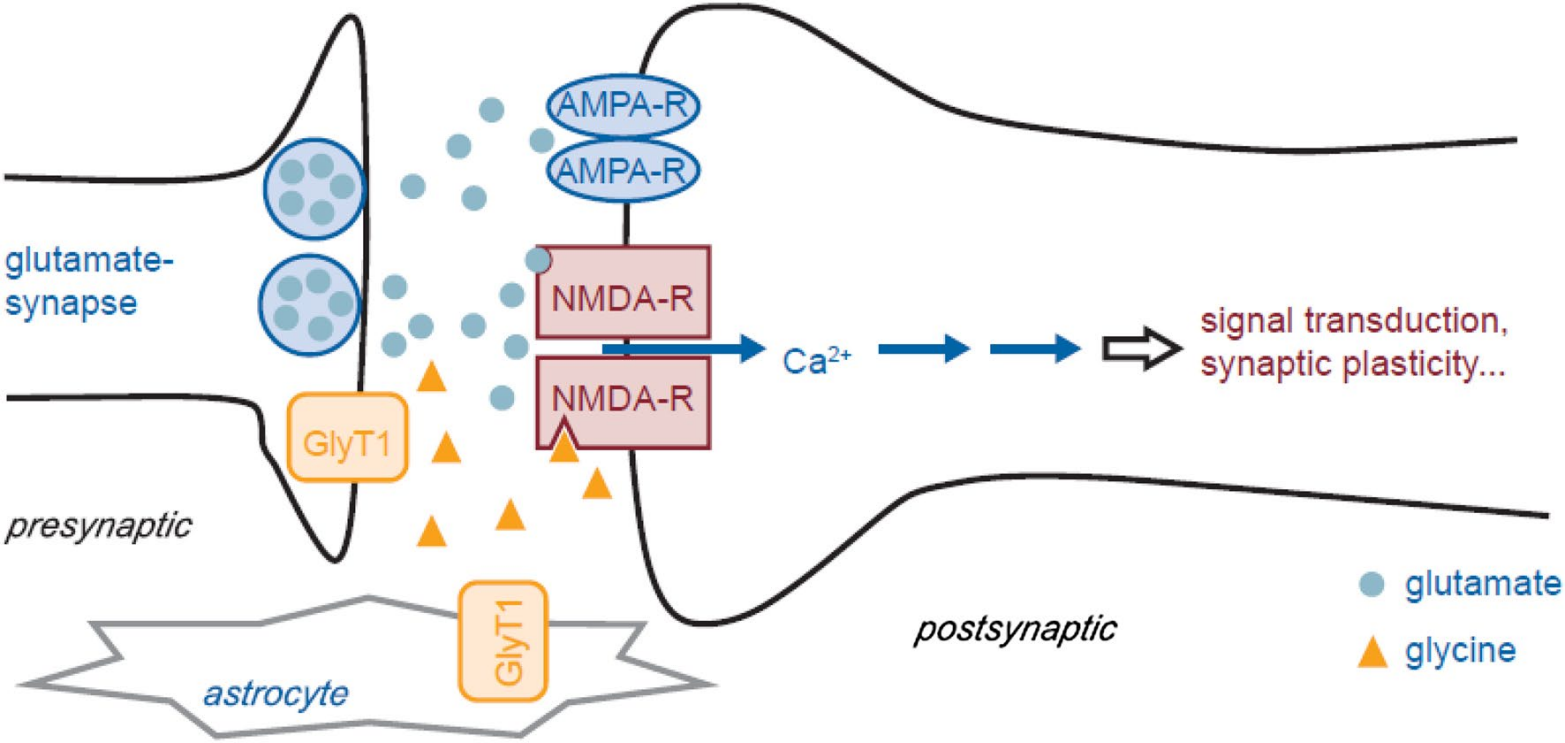
Iclepertin mode of action. *AMPA-R* α-amino-3-hydroxy-5-methyl-4-isoxazolepropionic acid receptor, *Ca*^*2+*^ calcium, *GlyT1* glycine transporter 1, *NMDA-R* *N*-methyl-D-aspartate receptor. Figure reused with permission from Moschetti V et al. (2016) Br J Clin Pharmacol 82(5):1315–24. 10.1111/bcp.13060

Glutamate signals are detected postsynaptically, mainly by two types of ligand-gated ion channels at the postsynaptic neuron; these include α-amino-3-hydroxy-5-methyl-4-isoxazolepropionic acid (AMPA) receptors as well as NMDA receptors [16, 17, 23]. Upon an action potential, glutamate is released presynaptically [18] and first binds to the AMPA receptors and forms a channel for the influx of sodium ions into the postsynaptic neuron [17, 23] (Fig. 2). This causes the postsynaptic neuron to depolarise and triggers an action potential in the postsynaptic neuron, which travels down the axon and transmits an excitatory signal to other neurons [18]. Due to the depolarisation triggers, magnesium ions dissociate from NMDA receptors [17, 23, 24] and then, accompanied by binding of glutamate and glycine to the receptor, the NMDA receptor channel opens [24]. Afterwards, both calcium and sodium ions move into the postsynaptic neuron [17, 23]. This postsynaptic calcium signal further triggers intracellular signalling pathways that increase the likelihood of a postsynaptic response to subsequent signalling events [17, 23]. This increased response to subsequent events is known as synaptic plasticity and is believed to play a key role in learning and memory [24].

NMDA receptors also provide excitatory input to inhibitory gamma-aminobutyric acid (GABA)-ergic interneurons and reciprocal signalling between excitatory glutamatergic neurons. GABA-ergic interneuron excitation is crucial for cortical network function and excitatory/inhibitory (E/I) balance within the network. This supports the generation of coordinated neural oscillations and overall neural network synchronicity, which are vital for cognitive function and sensory processing [25, 26]. For example, gamma oscillations in the prefrontal cortex are especially important for processes relating to working memory and are thought to be influenced by NMDA receptors within this E/I balance network [3, 26, 27].

In patients with schizophrenia, prolonged hypofunction of NMDA receptors may lead to impaired synaptic plasticity and, thus, impaired cognitive functioning in patients [16]. Changes in NMDA receptor function in different regions of the brain may cause varying symptoms; while alterations in NMDA function in the prefrontal cortex may lead to changes in cognition, alteration in NMDA function in the hippocampus may be linked to the development of psychosis [25]. Beside the key role of the NMDA receptor function for synaptic plasticity, the mechanisms underlying the effects of NMDA receptor hypofunction in CIAS are hypothesised to be attributable to reduced excitatory input to NMDA receptors located on GABA-ergic inhibitory interneurons in cortical brain areas such as the prefrontal cortex. This leads to reduced functional inhibition of excitatory pyramidal neurons by interneurons, known as disinhibition of pyramidal cells, which causes an E/I imbalance and perturbed network function in the prefrontal cortex and therefore could explain cognitive dysfunction of patients with schizophrenia [26–28].

Indeed, it is widely reported that patients with schizophrenia present with quantifiable sensory and neural network disturbances, which can be assessed using neurophysiological parameters measured using electroencephalography (EEG) [26, 27, 29–32]. Furthermore, cortical network deficits, including disruptions of gamma oscillations, have been associated with cognitive processes, such as working memory and executive function [3]. For example, unlike healthy controls, patients with schizophrenia fail to increase gamma oscillatory power in the dorsolateral prefrontal cortex during working memory tasks [27].

The role of NMDA receptor hypofunction in disrupted network processing and CIAS is also supported by the effects of the non-competitive NMDA receptor antagonist ketamine [33]. When administered in healthy individuals, ketamine produces psychosis and symptoms similar to schizophrenia, including executive and cognitive impairment [33]. Furthermore, NMDA receptor antagonists have been reported to induce deficits in sensory processing and neural network connectivity in both animal models and healthy volunteers [34–37].

Evidence from studies that modulate glutamate via other pathways also supports targeting of glutamatergic signalling to improve cognition; for example, inhibitors of D-serine and D-aspartate (D-amino acids that play key roles in the pathogenesis of schizophrenia) have been used to successfully address CIAS [38]. Specifically, sodium benzoate, a D-amino acid oxidase inhibitor, when administered at a dose of 2 g per day as an add-on to clozapine treatment, improved positive and negative symptoms as well as quality of life measures in patients with schizophrenia compared with those treated with placebo [39]. Another D-amino acid oxidase inhibitor, luvadaxistat, is currently being investigated as a potential treatment in the ongoing ERUDITE trial (NCT05182476) [40] following positive efficacy results in CIAS but not in treating negative symptoms in the INTERACT study (NCT03382639) [41]. Overall, given the evidence for the role of NMDA receptor hypofunction in CIAS, it can be hypothesised that increasing synaptic glycine levels by GlyT1 inhibition could normalise NMDA receptor hypofunction in patients with schizophrenia and thus facilitate glutamatergic signalling and synaptic plasticity. This would normalise NMDA receptor-mediated E/I imbalance in the cortex, which ultimately should lead to improvement of cognition in patients [22]. Therefore, inhibitors of GlyT1, which block the reuptake of glycine, could offer a promising treatment approach for CIAS [42, 43] (Fig. 2).

## GlyT1 inhibitors in preclinical and clinical studies

A number of GlyT1 inhibitors have been developed over the last 10 years to address CIAS. Sarcosine, reviewed elsewhere, was investigated in clinical trials assessing effects on symptom severity compared with placebo and demonstrated superior efficacy in reducing positive and negative symptom severity in specific subgroups of schizophrenia (chronic and non-treatment refractory disease), but its efficacy in relation to cognitive symptoms remains unclear [44–46].

Bitopertin, a non-competitive GlyT1 inhibitor, has also been investigated as a potential treatment for schizophrenia symptoms. A Phase II trial in patients with dominant negative symptoms demonstrated modest improvements in negative symptoms following bitopertin treatment versus placebo as an add-on to standard of care therapy [47]. However, in subsequent Phase III confirmatory trials, treatment with bitopertin alone did not result in significant improvements in positive and negative symptoms as assessed using the positive and negative syndrome scale [48–50].

The more recently emerging PF-03463275 is a competitive GlyT1 inhibitor currently undergoing Phase II trials [51]. When administered in a range of doses from 10 to 40 mg in patients with schizophrenia and healthy controls, PF-03463275 enhanced neuroplasticity in patients with schizophrenia and improved working memory accuracy when tested on healthy subjects. However, PF-03463275 administration did not abrogate ketamine-associated alteration in accuracy or reaction time [51]. Interestingly, analysis of the pooled effects of PF-03463275 on long-term potentiation across all doses in schizophrenia patients (10–60 mg) demonstrated a peak effect at the 40 mg dose (~ 75% GlyT1 occupancy), displaying an inverted ‘U’ dose–response profile [51], suggesting that dose selection and GlyT1 receptor occupancy are important for GlyT1 inhibitor efficacy. These positive effects on neuroplasticity support further investigation of GlyT1 inhibitors in the treatment of CIAS and emphasise the importance of optimal dose selection in clinical trials.

Following disappointing results in clinical trials with sarcosine and bitopertin, this review aims to summarise the most recent progress made in this field, and specifically the potential for the novel potent and selective GlyT1 inhibitor iclepertin to address cognitive symptoms in schizophrenia; to date, studies evaluating GlyT1 inhibitors have not targeted cognitive symptoms specifically.

## Development of the GlyT1 inhibitor iclepertin (BI 425809)

### Overview

**Fig. 3:**
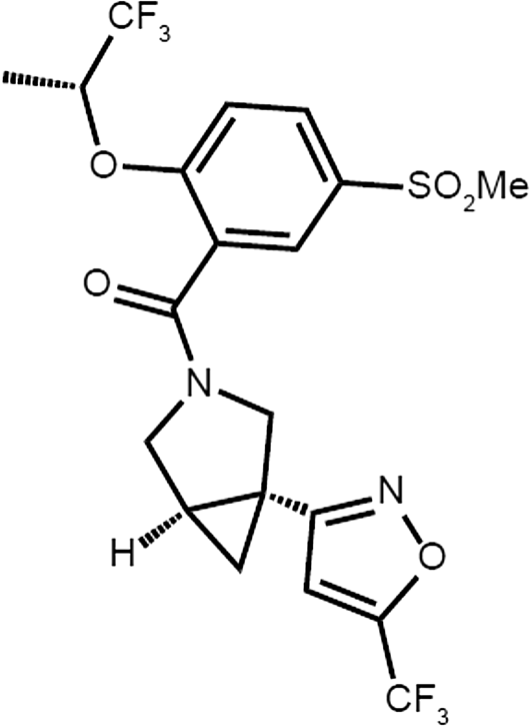
Chemical structure of iclepertin. Figure reused with permission from Rosenbrock H et al. (2022) J Pharmacol Exp Ther 382(2):223–32. https://doi.org/10.1124/jpet.121.001071. Wiley (Publisher)

Iclepertin (BI 425809) is a novel potent and selective GlyT1 inhibitor, currently under development by Boehringer Ingelheim for the treatment of CIAS (Fig. 3). The safety, pharmacokinetic (PK) profile, and efficacy of iclepertin has been investigated in several Phase I and II clinical trials since 2014 [52–55]. Results thus far have demonstrated iclepertin to be both safe and well tolerated in healthy individuals, as well as in patients with schizophrenia [52–55]. Furthermore, non-clinical studies in rodents and a Phase II clinical trial in patients with schizophrenia have demonstrated pro-cognitive effects of iclepertin [52, 56]. Phase III clinical trials are currently underway to confirm these positive findings and to investigate the possible benefits of iclepertin in patients with CIAS. Here we review the clinical development of iclepertin to date.

### Non-clinical studies of iclepertin

#### Evidence for target engagement

**Fig. 4:**
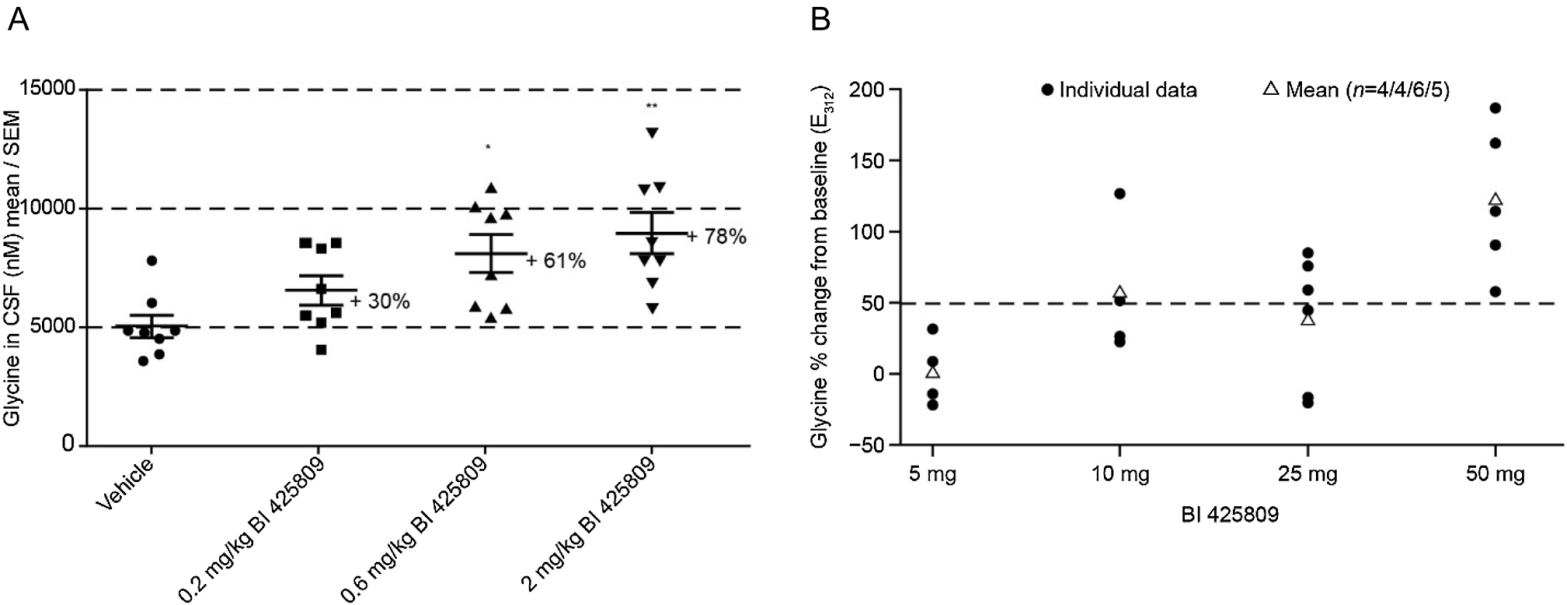
**a** Effects of BI 425809 on glycine levels in rat cerebrospinal fluid (CSF)* and **b** Percentage change from baseline in individual and mean E_312_ of glycine in CSF after multiple dosing of BI 425809 in healthy male subjects^ꝉ^. *Data are expressed as mean ± SEM of eight animals per group; *p < 0.05, **p < 0.01 as compared with vehicle, Dunnett’s post hoc analyses. ^ꝉ^The dotted line represents a 50% increase from baseline in CSF glycine concentration E_312_, effect of glycine at the time point 312 h before the last dose is given. *CSF* cerebrospinal fluid, *SEM* standard error of measurement. Figures reused with permission from Rosenbrock H, Desch M, Kleiner O et al. (2018) Clin Transl Sci 11:616–23. https://doi.org/10.1111/cts.12578, Wiley (Publisher)

Central target engagement, i.e. inhibition of GlyT1 in the brain, was assessed by increase of glycine levels in the cerebrospinal fluid (CSF) of rodents [57]. The results of that study demonstrated that a single oral administration of iclepertin caused a dose-dependent increase in glycine CSF levels of rats [57] (Fig. 4a). Further data analysis demonstrated that CSF glycine increased by 50–60% at iclepertin CSF concentrations in the range of the GlyT1 half-maximal inhibitory concentration (IC_50_) (5.0 nM in human SK-N-MC cells) [57]. This dose-dependent increase in glycine CSF levels, as shown in Fig. 4a, indicated the functional target engagement of GlyT1 in the brain and confirmed the use of glycine analysis in CSF as a suitable translational approach for central target engagement in humans [57]. In rats, iclepertin administered orally at doses ranging from 0.2 to 1.8 mg/kg improved social memory performance at all tested doses [56] and correlated with expected CSF iclepertin levels of 0.7–7-fold of the GlyT1 IC_50_ on GlyT1 (5.2 nM) during the testing [57].

#### Cortical network function and cognitive effects of iclepertin

To investigate further the pharmacology of iclepertin, its effects in rodents on sensory processing, cortical network function and cognitive performance, including working memory and social recognition, were assessed [56]. Briefly, the NMDA receptor antagonist MK-801 was used to induce cortical network deficits (E/I imbalance), which were then measured by EEG to determine the effects of iclepertin on both sensory processing and cortical network function. Analysed EEG parameters included N1 (also known as N100), a negative potential caused by a sensory stimulus, auditory event related potentials, and auditory steady-state response (ASSR). Results demonstrated that iclepertin attenuated MK-801-induced deficits in N1 amplitude and N1 gating, as well as on the 40 Hz ASSR, which supports the effects of GlyT1 inhibition by iclepertin on cortical network function [56]. Furthermore, evidence for pro-cognitive efficacy of iclepertin on memory function was demonstrated in this study. For instance, iclepertin was shown to reverse MK-801-induced deficits in working memory in the spontaneous alternation task in mice, and to improve episodic memory function in rats, as assessed using the social recognition test with a 24-h forgetting paradigm [56].

### Phase I clinical trials of iclepertin

The first-in-human Phase I, two-part, single-site trial of iclepertin (NCT02068690) tested the effects of single doses of iclepertin in liquid formulation at 0.5 mg, 1 mg, 2 mg, 5 mg, 10 mg, 25 mg, 50 mg, 100 mg and 150 mg. Of note, the liquid formulation used in this trial differed from the tablet formulation used in subsequent trials and showed greater exposure, as demonstrated by higher T_max_, C_max_ and AUC (area under the curve) compared with corresponding doses of the tablet formulation [53]. Results from this trial demonstrated single doses of iclepertin in liquid formulation to be safe and well tolerated in the dose range predicted to be clinically relevant (10–25 mg), with a favourable PK profile in healthy male volunteers [53]. Central nervous system and visual effects were the most common adverse events (AEs) reported from this initial trial. Incidences of AEs were found to be dose dependent, with an increase in nervous system disorders, eye disorders, and ear or labyrinth disorders occurring at the 100 mg and 150 mg dose levels [53]. However, it is important to note that there were no dose-dependent changes in laboratory parameters, including hormone levels, haematology findings or vital signs [53].

Thereafter, a Phase I, single-site, two-part trial (NCT02337283) investigated multiple ascending doses of once-daily iclepertin 10–75 mg tablets in young healthy male and female volunteers aged 18–50 years, and doses of 25 mg or 50 mg in elderly healthy volunteers aged 65–80 years [54]. No serious AEs were reported in this study, and all treatment emergent AEs were of mild or moderate intensity [54]. In young volunteers, nervous system disorders and infections were the most commonly reported AEs, while among the elderly group, gastrointestinal disorders and fatigue were most common. However, there were no clinically relevant differences in the profile of AEs between treatment groups [54]. Overall, multiple ascending doses of once-daily iclepertin were found to be safe and well tolerated at dose levels of 10–75 mg in healthy young volunteers, and 25–50 mg in elderly volunteers [54]. Twice-daily doses of 75 mg were also safe and well tolerated in young healthy volunteers [54].

An additional Phase I trial (NCT02362516) investigated the PK profile, pharmacodynamics, and safety and tolerability of multiple oral doses of 5 mg, 10 mg, 25 mg or 50 mg dose levels in 25 healthy male volunteers [57]. Results from this trial demonstrated a very good correlation between plasma and CSF concentrations; with increasing iclepertin dose, the exposure to iclepertin in plasma and in CSF also increased, leading to a dose-dependent increase in glycine CSF levels [57] (Fig. 4b). Although the IC_50_ was reached even with the 5 mg dose, no statistically significant effect on CSF glycine levels was observed at this lowest dose, which may be accounted for by intra-individual variability and small group size [57]. The results of this trial provide further evidence for the functional target engagement of GlyT1 by iclepertin as well as support for a potential therapeutic mechanism for CIAS [57].

To investigate any potential racial differences in the safety, tolerability and PK of iclepertin, a further Phase I trial (NCT02383888) was conducted in healthy male Chinese and Japanese volunteers, aged 20–45 years [55]. The results of this trial confirmed both the safety profile as well as the PK exposure levels to be consistent with previous trials in Caucasian volunteers [55].

Overall, results from Phase I clinical trials found iclepertin to be safe and well tolerated by healthy volunteers in single doses of up to 25 mg [53], and multiple doses of up to 75 mg once and twice daily (i.e. 150 mg per day) [54]. Taken together with data from non-clinical studies, these data provide support for the dose ranges and once-daily regimen selected for investigation in Phase II clinical trials [55, 56].

Pharmacological trials with iclepertin in healthy volunteers have been conducted to further understand the PK of iclepertin [58]. For example, drug–drug interaction trials of iclepertin in combination with a strong cytochrome P450 3A4 (CYP3A4) inhibitor (itraconazole) and inducer (rifampicin) have been published previously but were not considered of relevance to this review [58]. More PK studies of iclepertin will be performed to further understand the drug–drug interaction and tolerability.

### Phase II clinical trials of iclepertin

#### Efficacy and safety

The efficacy and safety of iclepertin for treatment of cognitive impairment in patients with schizophrenia was investigated in a Phase II, randomised, double-blind, placebo-controlled, parallel-group trial (NCT02832037) [52]. This trial was conducted across 81 centres in 11 countries and included 509 adult male and female patients with a Diagnostic and Statistical Manual of Mental Disorders 5th edition diagnosis of schizophrenia [52]. Patients were randomly assigned (1:1:1:1:2) to 2 mg, 5 mg, 10 mg and 25 mg dose groups or placebo. Patients were given the assigned treatment once a day for 12 weeks in addition to their stable antipsychotic treatment [52].

**Fig. 5:**
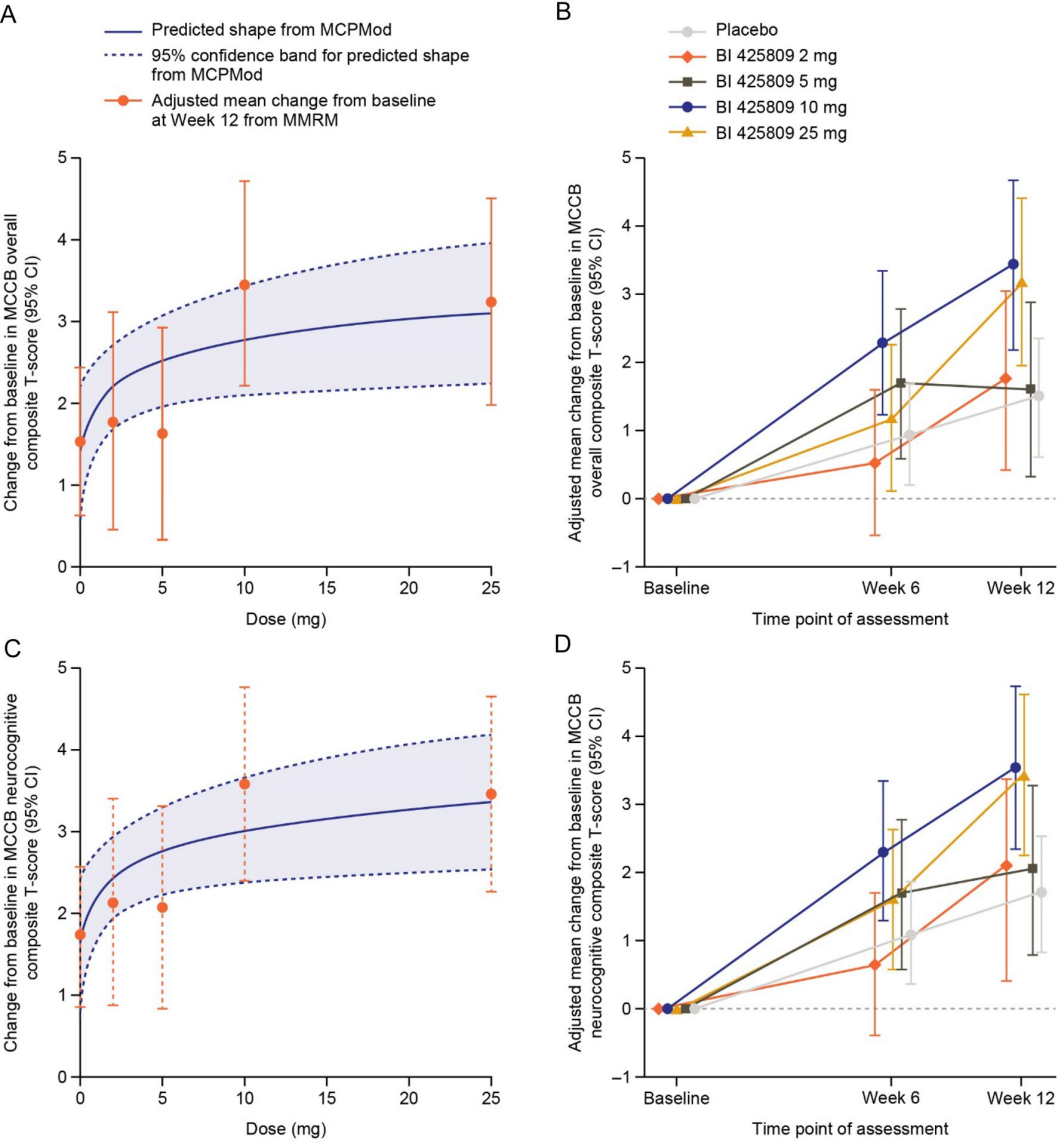
**a,** **b** Best-fitting dose–response curve and adjusted mean change from baseline for MCCB overall composite T-score; **c**,** d** MCCB neurocognitive composite T-score. *CI* confidence interval, *MCCB* Measurement and Treatment Research to Improve Cognition in Schizophrenia (MATRICS) Consensus Cognitive Battery, *MCPMod* multiple comparison procedure and modelling, *MMRM* mixed model repeated measures. Figure reused with permission from Fleischhacker, WW et al. (2021) Lancet Psychiatry 8(3): 191–201

The primary efficacy endpoint was the change from baseline in the MATRICS Consensus Cognitive Battery (MCCB) overall composite T-score at 12 weeks; the secondary efficacy endpoint was change from baseline in the interviewer-assessed Schizophrenia Cognition Rating Scale (SCoRS) [52]. A non-flat dose–response curve for change from baseline at Week 12 in MCCB overall composite T-score was demonstrated with treatment with an iclepertin dose of 2–25 mg, suggesting improvements in cognition compared with placebo [52]. Treatment with iclepertin 10 mg or 25 mg demonstrated the greatest improvements in cognition; however, the 25 mg dose did not provide any additional cognitive benefit compared with the 10 mg dose [52] (Fig. 5a, b). The efficacy at the 10 mg dose and the corresponding exposure levels were in the range of exposure levels achieving about 50% glycine increase when compared with the data of the phase 1 trial in healthy volunteers [52, 57]. Similarly, greater improvements in change from baseline in MCCB neurocognitive composite T-score with iclepertin 10 mg and 25 mg were observed compared with placebo at 6 weeks and 12 weeks (Fig. 5c, d). For the secondary efficacy endpoint (change from baseline in SCoRS interviewer-rated total score) at Week 12, no dose–response model was statistically significant, and there was no significant improvement in change from baseline compared with placebo in any treatment group [52].

Furthermore, analysis of iclepertin plasma concentrations and PK parameters showed a dose-dependent increase in exposure, though the increase was less than dose-proportional across the dose range of 2–25 mg [52]. All doses were found to be safe and well tolerated, consistent with previous Phase I trial data.

#### Iclepertin in combination with computerised cognitive training

A Phase II, multicentre, randomised, double blind, placebo-controlled, parallel-group trial (NCT03859973) combining once-daily iclepertin 10 mg together with at-home computerised cognitive training (CCT) in patients with schizophrenia is currently ongoing to investigate the potential dual effect of this combination therapy on CIAS [59]. The addition of CCT is being investigated in this trial as it is thought that pharmacotherapies targeting cognition may require concurrent cognitive stimulation to enhance potential cognitive effects [60]. Patients with schizophrenia may often have a low level of cognitive and environmental stimulation, which may prevent cognitive improvements from occurring with pharmaceutical interventions alone [60].

### Phase III clinical trials of iclepertin

**Fig. 6:**
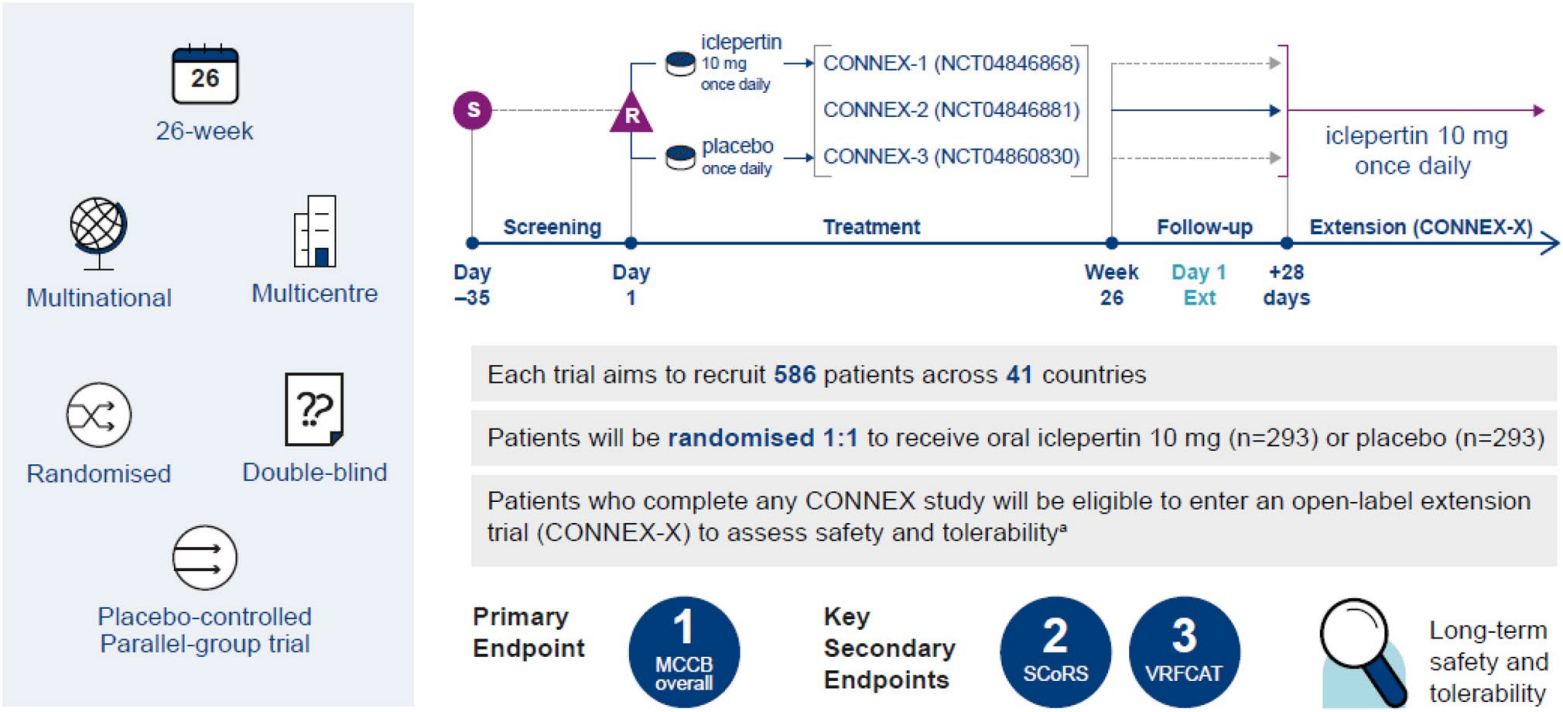
Study diagram for the ongoing Phase III trials (CONNEX 1/NCT04846868; CONNEX 2/NCT04846881; CONNEX 3/NCT04860830). Note: patients were stratified by screening MCCB score at randomisation. ^a^Patients from CONNEX-1 and 3 can go directly into the open-label study; patients from CONNEX-2 must complete discontinuation and follow-up first. Broken grey arrow, optional follow-up; solid blue arrow, must complete follow-up. MCCB, Measurement and Treatment Research to Improve Cognition in Schizophrenia (MATRICS) Consensus Cognitive Battery; R, Randomisation; S, Screening; SCoRS, Schizophrenia Cognition Rating Scale; VRFCAT, Virtual Reality Functional Capacity Assessment Tool

There are currently three multinational, Phase III trials underway, which are investigating further the efficacy and safety of iclepertin in improving cognition and daily functioning in a large cohort of patients with schizophrenia. These trials are: CONNEX 1/NCT04846868; CONNEX 2/NCT04846881 and CONNEX 3/NCT04860830, with an additional open-label safety extension trial, CONNEX-X (Fig. 6).

## Discussion

Cognitive symptoms, including impairments in working memory and executive function, are common in schizophrenia and often lead to impaired daily functioning in patients [3, 5, 31]. However, to date, there are currently no approved pharmacological treatments for CIAS [4]. NMDA receptor hypofunction resulting in the imbalance in excitatory and inhibitory signalling has been implicated as a potential cause for symptoms associated with schizophrenia, particularly cognitive impairment. Increasing synaptic glycine levels to enhance glutamatergic signalling by blocking the reuptake of glycine via inhibitors of GlyT1 represents a promising target for the treatment of CIAS.

Iclepertin, a potent and selective GlyT1 inhibitor, has been demonstrated to be safe and well tolerated in healthy volunteers and in patients with schizophrenia [52–55]. Non-clinically, iclepertin attenuated deficits in EEG parameters induced by the NMDA receptor antagonist MK-801 and demonstrated improved working and episodic memory performance in rodent cognition tasks at CSF concentrations in the range of approximately 1–6-fold of the IC_50_ [56]. Central target engagement of iclepertin was demonstrated by a dose-dependent increase of glycine in rat and human CSF, reaching 50% glycine increase at CSF concentrations of about 1-fold IC_50_ in rats and 2-fold IC_50_ in humans at the 10 mg dose, respectively [57]. Phase I data demonstrated that plasma exposure of single ascending doses of iclepertin increased dose proportionally [53] at the predicted therapeutic exposure range of iclepertin, but less than dose proportionally at ≥ 50 mg [54].

The non-clinical findings on pro-cognitive efficacy were supported further by Phase II data in patients with CIAS, which demonstrated significant improvements in cognition versus placebo at 10 mg or 25 mg of iclepertin, with no additional benefit noted from the 25 mg dose compared with the 10 mg dose [52]. Three Phase III trials are currently ongoing to confirm these initial safety and efficacy findings and to report on the long-term effects of iclepertin on daily functioning in patients. If Phase III trials are successful, iclepertin may become the first approved once-daily pharmacotherapy to effectively improve cognition, with the potential to alleviate an urgent unmet clinical need for patients with schizophrenia who may be living with the daily burden of CIAS.

## Data Availability

The manuscript does not contain clinical studies or patient data.
